# Histopathologic Profile of Alopecia Areata in Indian Patients

**DOI:** 10.4103/0974-7753.66906

**Published:** 2010

**Authors:** V Chaitra, T Rajalakshmi, R Kavdia

**Affiliations:** Department of Pathology, St. John’s Medical College, Bangalore, India

**Keywords:** Alopecia Areata, histopathology, non-scarring alopecia

## Abstract

**Background:**

Alopecia Areata (AA) is a “non-scarring” alopecia that has an autoimmune basis. Though clinically distinctive, problems arise in diagnosis depending on the temporal stage of the disease at presentation; some of them progress to scarring alopecia and predicting its prognosis is difficult. Histological changes depend on the disease stage and site of the biopsy.

**Objectives:**

To describe the spectrum of histologic features in AA.

**Materials and Methods:**

A prospective and retrospective study of H and E sections of all biopsies signed out as AA between 2001 and 2009 (20 cases) was undertaken.

**Results:**

The diagnosis was made on vertical sections in all cases. The total number of hair follicles ranged from 1 to 24 with an average of 7 and comprised mainly terminal follicles. Vellus follicles were scanty. Anagen to non-anagen ratio was 1:1.62. Miniaturization of follicles was noted in five (25%) cases. Peribulbar inflammation was seen in all the cases with a dominance of lymphocytes. Perifollicular fibrosis was noted in 12 (60%) and pigment casts in 5 (25%) cases. Scarring was seen in two cases. In these cases, a diagnosis of AA was rendered on the basis of even spacing of the fibrotic units and remnants of the catagenic basement membrane within the scars. The epidermis and interfollicular dermis were normal in all the cases.

**Conclusion:**

The most consistent features of AA are an increase in non-anagen terminal follicles and peribulbar lymphocytic infiltrate. The etiology can be determined even in cases that have progressed to scarring.

## INTRODUCTION

Alopecia areata (AA) is the commonest cause of non-scarring alopecia. It is thought of as an organ-specific autoimmune disorder with a genetic predisposition.[1] Patients of AA present with patchy, abrupt hair loss and thinning of the hair follicle at the isthmus giving the characteristic appearance of ‘exclamation point hairs.’ Though clinically distinctive in most instances, AA needs to be differentiated from Telogen effluvium, androgenetic alopecia and Trichotillomania.[1] Generally, AA is classified as non-scarring. But, there are several reports of the disease progressing to scarring and leading to permanent hair loss.[2] In such instances, the pathologist has a pivotal role to play in establishing the correct diagnosis. The histologic changes vary depending on the stage of the disease and most pathologists have limited experience with their interpretation, as classic AA is rarely biopsied. There is no study that describes the microscopic changes of AA in Indian patients. This prompted us to revisit the histopathology of AA.

### Objectives

To describe the histopathologic features noted in AA.

## MATERIALS AND METHODS

This was a study carried out for 2 years prospectively (2008–2009), along with a retrospective review of cases from 2001 to 2007. This included all cases signed out as AA at the Department of Pathology. Biopsies were obtained using a 4-mm punch in all cases. A minimum of 15 H and E sections were studied in all cases. The initial diagnosis was based on predominance of non-anagen terminal follicles, infiltrates in the deeper/peribulbar portion of the follicles and absence of significant epidermal changes. The features particularly studied included: number of hair follicles (terminal, vellus), number of anagen, catagen and telogen follicles, miniaturization, dermal inflammation (location and nature), perifollicular inflammation (location and nature), spacing of follicular units, pigment casts, scars, perifollicular fibrosis, sebaceous glands and interfollicular dermis. In those cases that had scarring, Alcian blue–Periodic Acid Schiff was done. Clinical data were retrieved from the patients’ case files.

## RESULTS

Over 9 years, there were 20 cases of AA that were biopsied. The age ranged from 9 to 42 years with a slight female predominance (F:M=1.5:1). The duration of the lesions at the time of biopsy ranged from 3 months to 5 years. Hair loss was patchy in 18 cases and diffuse in 2 cases. One patient had a family history of similar lesions.

**Figure 1:**
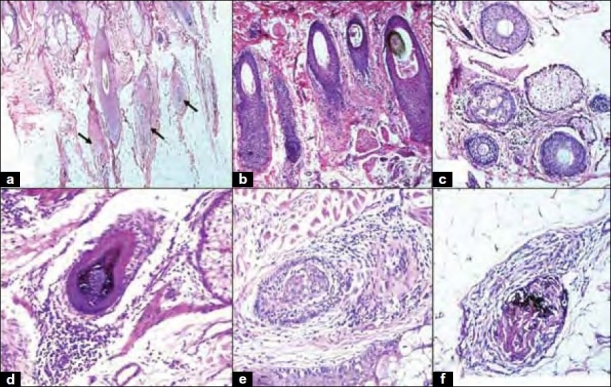
(a) Predominance of non-anagen terminal follicles (H and E, ×40); (b) catagen follicles that appear to be in the same phase (H and E,×100); (c) miniaturization of follicles (H and E, ×100); (d) peribulbar lymphocytic infiltrates (H and E, ×200); (e) perifollicular fibrosis and inflammation (H and E, ×100); (f) melanin casts within fibrotic areas (H and E, ×100)

**Figure 2:**
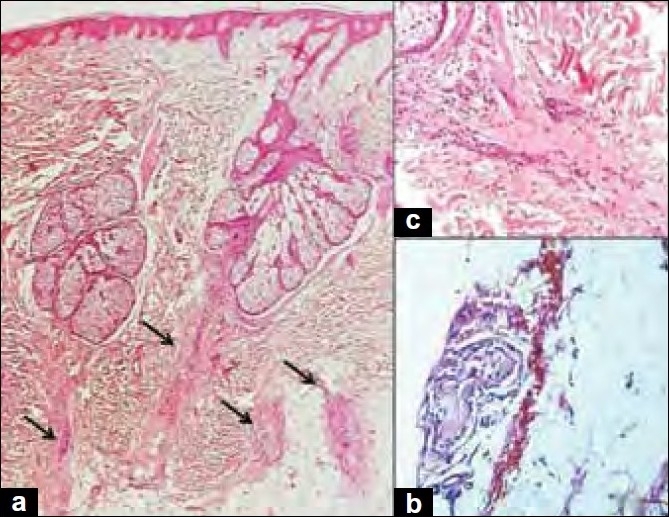
(a) Evenly spaced scars with normal epidermis and intervening dermis (H and E, ×40); (b) lymphocytic infiltrates in the deeper portions of the scars (H and E, ×100); (c) remnants of the wrinkled catagenic basement membrane with a basophilic tinge (H and E, ×100)

All biopsies had vertical sections and five of them in addition had transverse sections. The total number of hair follicles ranged from 0 to 24 with an average of 7 per section and constituted mainly terminal follicles. There was no relation of these factors with the age or gender of the patients. Vellus follicles were scanty. Non-anagen follicles, that is, follicles in catagen [Figure 1a] and telogen phase were predominant. All catagen follicles appeared to be in the same phase [Figure 1b]. Anagen to non-anagen ratio was 1:1.62. Miniaturization of follicles was noted in five (25%) cases [Figure 1c]. Peribulbar inflammation was seen in all the cases [Figure 1d]. Lymphocytes were the predominant cell type in the infiltrate. Perifollicular fibrosis was noted in 12 (60%) [Figure 1e] cases, pigment casts [Figure 1f] in 5 (25%) cases. Scarring was seen in two cases. In these cases, a diagnosis of AA was rendered on the basis of a normal epidermis, even spacing of the fibrotic units [Figure 2a], lymphocytic infiltrates in the deeper portions [Figure 2b] and remnants of the catagenic basement membrane [Figure 2c]. The epidermis, rest of the dermis and sebaceous units were unremarkable. None of the biopsies demonstrated perivascular or peri-eccrine inflammation. The diagnosis was established on vertical sections in all the cases.

## DISCUSSION

AA is a clinically distinctive form of ‘non-scarring’ alopecia that is rarely biopsied and hence, many pathologists are unfamiliar with its interpretation. In addition, literature stresses that transverse sections are a must for establishing a correct diagnosis, which are not done in most laboratories.[3] The role of a pathologist is vital when dealing with atypical presentations, such as patients progressing to scarring, use of topical medications that alter the picture and of late, in trying to provide prognostic information.

There have been two studies from India on AA which have described only the clinical features and its association with other autoimmune diseases. There are no studies addressing the histopathologic profile of Indian patients.[4,5]

The histopathologic features vary depending on the stage of the disease, classified as early active and chronic phases.[3] The earliest and most important feature in the acute phase is the peribulbar lymphocytic infiltrate, which may also extend into the epithelium and hair matrix.[3,6] This was the most consistent finding even in our study. In later stages, the inflammation may be diminished or absent. In such cases, some authors have relied on the presence of eosinophils near hair bulbs and fibrous tracts.[1,7] Eosinophils were either absent or inconspicuous in all our cases and did not corroborate earlier findings.

A preponderance of non-anagen follicles, that is, catagen and telogen follicles, was seen in all 20 cases. All the catagen follicles within a biopsy were in a similar phase, that is, either early or late catagen. The anagen:non-anagen ratio was 1:1.62. Many centers depend on decreased anagen:telogen ratios on transverse sections.[1] It is difficult to distinguish catagen and telogen follicles at times and it is better to simply count them as non-anagen follicles. One may also see nanogen hairs, that combine characteristics of both catagen and telogen follicles.[3] In our study, we appreciated these changes on vertical sections alone. The transverse sections that were additionally done in five cases either showed similar features or were not contributory. The extent and location of perifollicular inflammation could not be assessed in the transverse sections. Therefore, we feel that vertical sections more than suffice for making this diagnosis. Transverse sections are technically demanding and their interpretation requires an in-depth knowledge of the transverse anatomy of the scalp, which most pathologists are not conversant with.

Miniaturization of follicles was observed in five of our cases. These are usually seen later in the course of the disease.[3,6] Clinically, there was no suspicion of androgenetic alopecia in these patients. We did not find an increase in vellus hairs, unlike some prior reports.[6] Pigment casts were seen in the fibrous tracts surrounding as well as within follicles in 25% of cases. This feature requires distinction from trichotillomania, which in addition shows broken, distorted hair shafts, trichomalacia and a remarkable absence of inflammatory cells. Perifollicular fibrosis was noted in 60% of cases, mirroring the chronicity of the disease.

AA is regarded as a non-scarring alopecia. However, the course of the disease is very unpredictable and upto 25% of patients progress to irreversible hair loss and scarring.[2] Two cases in the present series showed scarring, with a near-complete absence of terminal follicles. The follicular scars in these cases were evenly spaced with a mild lymphocytic infiltrate in the deeper portions of the scar. The epidermis and interfollicular dermis were normal. There was no increase in dermal mucin or peri-eccrine inflammation. These features aided in differentiation from alopecia of lupus erythematosus, which can also show peribulbar inflammation. Lichen planopilaris, the other common etiology for scarring alopecia which can also have evenly spaced scars, additionally demonstrates changes of interface dermatitis and preferential location of lymphocytes around the isthmic and infundibular portions of the follicle, as opposed to a deeper/peribulbar location in AA. In late stages, there is an absence of inflammatory infiltrate in both diseases and distinction may be difficult. A notable feature in this study was the presence of remnants of the catagenic basement membrane (with a basophilic hue) both within the scars and elsewhere in the sections, which has not been stressed before, though it has been described by Dr. Ackerman.[8] In the complete absence of follicles and inflammatory cells, this feature will point to the etiology. We hope that larger studies will validate our findings. Preponderance of miniaturized follicles and persistence of peribulbar inflammation are said to indicate incomplete recovery.[9]

In summary, pathologists can make a confident diagnosis of AA based on peribulbar lymphocytic infiltrates, predominance of non-anagen terminal follicles, pigment casts and perifollicular fibrosis with a normal epidermis and interfollicular dermis. Even in cases that have progressed to scarring, it is possible to elicit the primary cause. Routine vertically embedded sections of scalp biopsies are more than adequate to ascertain the diagnosis.

Although the role of histopathology as a prognostic tool in AA is unclear, it is expected to assume greater importance with newly emerging drugs and biologic therapies.[9] It is accordingly essential for us to be cognizant of the gamut of features and record them to serve as a baseline for future studies.
